# Longitudinal Analysis of Marathon Runners’ Psychological State and Its Relationship With Running Speed at Ventilatory Thresholds

**DOI:** 10.3389/fpsyg.2020.00545

**Published:** 2020-03-27

**Authors:** Eneko Larumbe-Zabala, Jonathan Esteve-Lanao, Claudia A. Cardona, Alberto Alcocer, Alessandro Quartiroli

**Affiliations:** ^1^Sport Psychology Private Practice Tenerife, Canary Islands, Spain; ^2^AIYM Training System, Mérida, Mexico; ^3^Departamento de Ciencias de la Salud, Universidad del Valle de México, Mérida, Mexico; ^4^ERGO Exercise Performance Assessment Laboratory, Mérida, Mexico; ^5^Department of Psychology, University of Wisconsin-La Crosse, La Crosse, WI, United States

**Keywords:** marathon, running, self-efficacy, motivation, anxiety, fitness, ventilatory thresholds, running economy

## Abstract

Psychological variables such as motivation, self-efficacy, and anxiety have been widely studied in marathon runners, usually within the framework of Bandura’s theory of self-efficacy. It is also assumed a link between self-perceived fitness and physiological performance parameters such as speed at ventilatory thresholds and running economy. The purpose of this paper is to describe longitudinal trends of self-perceptions and examine their link to physiological performance parameters over time. Sixteen healthy recreational marathoners (8 males and 8 females), aged *M* = 37.6 (*SD* = 3.9) who were about to participate in a major marathon agreed to participate. After 3 months of regular training and competition in shorter distances, all participants trained during a 16-week macrocycle under the supervision of the same coaching staff. At 4-week intervals, the participants responded five times the Podium questionnaire, measuring self-perceived psychological state relative to the upcoming race, and performed five exercise performance parameters tests. Linear mixed-effects models were used to analyze the trends and associations. In general, Podium questionnaire scores were within the standard range, with the lowest values at the beginning and the highest values closer to race day. Although only perceived fitness (*p* < 0.001, Cohen’s *f*^2^ = 1.19) and somatic anxiety (*p* < 0.001, *f*^2^ = 0.32) showed large effect sizes for the whole longitudinal period, other partial increases were found between time points. All physiological performance parameters presented significant improvements over time (Aerobic Threshold speed, *p* < 0.001, *f*^2^ = 1.04; Anaerobic Threshold speed, *p* < 0.001, *f*^2^ = 0.498; Running Economy in VO_2_, *p* < 0.001, *f*^2^ = 0.349; Running Economy in energy, *p* = 0.024, *f*^2^ = 0.197). The analysis of changes between consecutive time points revealed that improving perceived physical condition predicted improving self-efficacy (*p* < 0.001, *f*^2^ = 1.33), and improvements in motivation were predicted by improvements in either self-efficacy (*p* < 0.001, *f*^2^ = 0.36) or perceived physical condition (*p* = 0.003, *f*^2^ = 0.17). Improvements in perceived fitness, self-efficacy and motivation were associated with small effect-size improvements (decreases) in anxiety. None of the physiological performance parameters was shown to predict changes in psychological variables, although their general trends over time correlated. The results have practical implications for sport psychologists and running coaches, supporting the need for integrated working.

## Introduction

The worldwide participation rate in recreational running peaked in 2016 and slightly declined since then (Andersen, 2019). Nevertheless, the number of worldwide marathon finishers was estimated to be 1,298,725 in 2018 (Andersen and Nikolova, 2019). Consequently, there has also been a growing interest in running events from the applied field of sport psychology (e.g., Meijen et al., 2017; Larumbe-Zabala et al., 2019).

Much of the sport physiology literature extensively descried the physiological variables related to marathon performance (Joyner, 1991, 2017). Among other factors, finishing time is particularly associated to Aerobic Threshold (AeT) and Anaerobic Threshold (AnT) speeds. Running Economy (RE) has also been identified as a determinant of better performance in long distance running. Consequently, it is expected from a marathon-specific training program to pursue the athlete’s optimal AeT and AnT speeds, and RE.

Performing well in shorter distances is also a strong predictor of physical condition and finishing times in longer distances (i.e., marathon). However, typically all training programs for the marathon distance limit the number of competitions the runners are advised to run during the season (Cardona et al., 2019). The reasons include controlling the effects of fatigue from the competition, and the need of concentrating the training program specifically on the marathon distance. The competition-induced fatigue can produce damaging effects, which explains why marathon runners do not cover the full distance during the preparation. Even though the aforementioned physiological variables interplay to determine running performance (finishing time), only tracking physiological variables over time would really reflect the fitness of a runner during the preparation (Jones, 2006).

From a psychological perspective, Bandura’s (1977) theory of self-efficacy has been very extensively used for investigating the influence of psychological constructs on sport and motor performance. This author defined self-efficacy as people’s beliefs about their capabilities to produce effects. It is assumed that high physical self-efficacy, together with other physical performance indicators, is a good predictor of finishing time and race performance of runners (e.g., Okwumabua, 1985; Gayton et al., 1986; LaGuardia and Labbé, 1993).

According to Bandura’s theory, expectations of personal efficacy are based on four sources of information: performance accomplishments, vicarious experience, verbal persuasion, and emotional arousal (Bandura, 1977, p. 195). As a consequence, athletes’ perceived improvement in physiological performance and running technique, as well as other factors such as experience, tactical skills, and self-regulation ability should be linked to higher perception of self-efficacy. Bandura (1997) made a clear distinction between self-efficacy and confidence. The former refers to the belief in one’s agentive capabilities that one can produce given levels of attainment, while confidence is a colloquial term.

Beliefs of self-efficacy also play a key role in the self-regulation of motivation (Bandura, 1994). Anxiety has been shown to be inversely correlated with self-efficacy (e.g., LaGuardia and Labbé, 1993), while positive correlations have been found between motivation and self-efficacy (e.g., Martin and Gill, 1995). In agreement with Bandura’s (1977) self-efficacy theory, qualitative studies have shown that high self-efficacy and motivation, and low anxiety characterize an optimal psychological state in marathon runners (Larumbe Zabala et al., 2009). However, both the normal and the optimal quantitative levels in the above-mentioned psychological variables to compete in a marathon still remain unexplored.

The majority of the studies measuring psychological variables are assessed cross-sectionally, as close as possible to the competition day. However, the theory often includes cause-effect relationships that are developed in the long-term. Despite psychological practice also demonstrates that psychological processes develop over time, and involve an intricate system of constructs, there is still lack of studies using longitudinal designs. As an example, a quick search on PsycInfo database between June 2009 and July 2019 for the term “sport psychology” yielded 5,621 academic publications. Among these items, only 245 contained the word “longitudinal” in any search field. Despite this information does not properly constitute a systematic review on the topic, one can assume that among a good number of the recent sport psychology-related studies only approximately 4.5% potentially involved longitudinal designs.

Consequently, the first aim of this study is to examine the longitudinal patterns of the psychological state of a group of standard recreational marathon runners, over the last 16-week macrocycle prior to competing in a race. If changes in motivation, self-efficacy, perception of physical fitness, or anxiety are detected, we hypothesize that anxiety will correlate negatively with the other psychological variables, based on inverse correlations shown in previous studies (Larumbe et al., 2015). Additionally, we also expect a high and positive correlation among motivation, self-efficacy, and perception of physical fitness according to Bandura’s theory of self-efficacy.

The second aim is to investigate to what extent changes in physiological measurements are positively correlated with changes in perceived physical fitness. More specifically, we will use indicators of physiological performance in marathon such as RE tests, and AeT and AnT speed tests as predictors of self-perceived physical condition. Our hypothesis is that increases in perceived physical fitness will positively correlate with improvements in the physiological tests. Since self-efficacy is expected to improve in part due to improving physical fitness, we also expect to find a high correlation between self-efficacy and perceived physical fitness, and implicitly also with the physiological variables.

## Materials and Methods

### Participants

Sixteen recreational runners, 8 males and 8 females, according to the current gender distribution of marathon runners described by Andersen (2019), aged *M* = 37.6 (*SD* = 3.9) who were to compete in a marathon and shared the same training group (location, program, and coaches) voluntarily consented to participate in this study. In order to ensure a homogenous sample of healthy recreational runners, inclusion criteria were: (a) to have been training for endurance running events at least during 1.5 years; (b) to show average physiological performance levels compared to recreational marathon runners; and (c) to show an average relative performance compared to marathon race winners. Exclusion criteria were: (a) to reveal any kind of coronary disorder via a stress test; (b) to miss more than three consecutive sessions by cause of an injury or disease; (c) to accomplish less than 90% of the training sessions specified in the program.

### Sample Size, Power, and Precision

Using G-Power software, a minimum sample size of 13 runners was calculated assuming a 0.05 significance level, a 0.80 power, and a correlation ≥0.70 among five repeated measures in a one-way ANOVA model, in order to detect moderate effect sizes (Cohen’s *f* ≥ 0.25). In order to prevent possible attrition, we increased the required sample size by 23% to 16 participants, and all of them eventually completed the entire study.

### Measures

#### Psychological Variables

The participants were asked to answer the Podium questionnaire (Larumbe et al., 2015). This questionnaire is composed of 20 items that measure the following scales of self-perceived psychological state relative to the upcoming race: motivation, self-efficacy, perceived fitness, perceived social support, somatic anxiety, and cognitive anxiety. The items are answered in visual analog scale (VAS) format of response, composed of two opposite words at the ends of a 100 mm line. The response is interpreted measuring the distance of the mark on each line with a ruler, and taking it as the score of each particular item (cf. Aitken, 1969). The reversed items were measured starting from the opposite end. The items were averaged within each of the six scales, to obtain final scores in a 0–100 range. Although the authors of Podium questionnaire used the term self-confidence to word the construct self-efficacy, the latter will be used in order to avoid confusion, as it better reflects the content of the factor.

#### Physiological Performance Assessments

A running performance graded test until voluntary exhaustion was conducted in order to identify Aerobic (AeT speed) and Anaerobic Threshold Speeds (AnT speed) and other descriptive variables such as VO_2__*max*_ and Maximal Aerobic Speed (MAS). These procedures were applied under lab conditions and have been extensively described elsewhere (Muñoz et al., 2014). A breath-by-breath system was used for gas exchange data collection (Med Graphics PFX Ultima, Medical Graphics Corporation, St Paul, MN, United States). Heart activity was continuously monitored by the integrated ECG system (CardioPerfect PRO, Welch Allyn Cardio Control BV, Delft, Netherlands) and a Heart Rate (HR) monitor (Polar RC3 GPS, Polar Electro Oy, Kempele, Finland). This graded test was conducted only during the first visit to the lab. As it has been previously reported, thresholds-associated HR determined during laboratory testing remains stable over the season despite significant improvements in the workload eliciting both thresholds (Lucía et al., 2000).

At first visit, a RE assessment was conducted through constant load treadmill bout of 15 min duration at a 1% grade and a selected speed according to the individual’s expected marathon pace (Jones and Doust, 1996). Once this speed was identified and used for the first assessment, it was kept constant through the remaining visits to the lab. Since it has been shown that RE calculations might be different in case of including Respiratory Quotient or considering O_2_ only (Fletcher et al., 2009), RE was calculated both as oxygen cost in mL/kg/km (REmL) and as energy cost in kcal/kg/km (REkcal) as described elsewhere (Fletcher et al., 2009). All these tests were conducted under the supervision of a sports medicine physician and the coaches.

A field test was regularly conducted on a 400 m synthetic track. Two 20 min bouts, interspersed by a 5 min passive rest, were conducted keeping the HR zones associated AeT and AnT speeds, respectively. Considering the HR kinetics and the potential HR drift, runners were instructed to reach the target HR not before the 3rd minute. Lap by lap (400 m), self-recordings were averaged to estimate updated field AeT and AnT speeds.

### Training Program

All sixteen participants were trained systematically, following a very similar training program with minor individual adaptations, in the same city, directly supervised by the same coaches, in order to compete in a major marathon on the same date. Before the specific marathon preparation, these runners followed 3–6 months of regular training focused on 10k and 21k competition distances.

A 16-weeks, specific marathon training macrocycle was conducted before the race. This program was divided in four mesocycles with 4 weeks each. The mesocycles had a 3:1 structure, so that the higher training load was promoted during the initial 3 weeks, followed by a 1-week reduced training load in order to facilitate recovery and supercompensation. Peak running training volume was set at week 11 while the longest distance run was conducted at the end of week 13 (21 days before the marathon race). This program structure was the same for all runners, except for minor adaptations of the total training volume performed on three separate subgroups (*n* = 5, 6, and 5) of runners according to their performance level (i.e., peak weekly training volume was between 80 and 100 km).

### Research Design and Procedures

The study followed a single-group longitudinal design, with five repeated measures along a 16-week macrocycle. Ethical approval was sought and granted by the European University of Madrid, Spain (CIPI/035/15). Participants were asked to sign an informed consent form for their enrollment in the study, and had to perform the study assessments (Podium questionnaire, lab RE and field AeT/AnT speeds assessments) on five occasions: at 116, 80, 60, 32, and 11 days prior to competing in the marathon. Each visit included a psychological test, field-test measurements, RE lab assessments and physiological tests as described above. All tests were conducted during the same approximate day time (field tests between 5:00 and 6:30 am; lab tests at the same time for each athlete) and under similar weather conditions (field tests under no rain, no wind, temperature 23–25°C, humidity 70–75%; lab tests under 21°C and 70% humidity), preceded by a 15 min easy jog 1–15 bpm below the ventilatory threshold, followed by a dynamic stretching workout.

### Statistical Analysis

Relative performance was calculated to compare men and women among the sample of marathon runners. For this purpose, each runner’s personal best time was divided by the average of the best times of the 10 fastest performers in the world, including only one performance per individual (cf. Deaner et al., 2011), which was 2:03:03 for males and 2:17:44 for females as of July 18, 2019 (International Association of Athletics Federations, 2019). Then these ratios were converted into percentages. Sample characteristics were summarized using mean (standard deviation) or median (interquartile range, IQR) for continuous variables and frequency (percentage) for categorical variables. Shapiro-Wilk test and histograms were used to assess normality of the distributions. To check differences between sexes, two-tailed Student’s *t*-test or Wilcoxon’s rank sum test were used as appropriate.

Podium questionnaire variables (motivation, self-efficacy, perceived fitness, social support, somatic anxiety, and cognitive anxiety) and running physiology variables (AeT speed, AnT speed, REmL, and REkcal) were summarized, plotted and overlaid using adjusted means and 95% confidence intervals (CI) after running linear mixed effects models, where time and participants were set as the fixed and random effects of the models respectively. Statistical significance of changes over time for all the dependent variables was assessed using (a) contrasts between baseline and subsequent time points, and (b) linear mixed effects models on the relative change from any given time point to the next, adjusted for participant random effects. Percent change for all above-mentioned variables was calculated as: (Measure_n_ – Measure_n__–__1_)/Measure_n__–__1_ × 100. Sidak correction was used to prevent multiple comparison error when necessary.

The association of physiological (AeT speed, AnT speed, REmL, and REkcal) with psychological variables (perceived physical fitness and self-efficacy from the questionnaire) over time was assessed using linear mixed effects models on raw values, and on percent change in both criterion and predictor variables adjusted for time as fixed effect and participant as random effect.

Residual maximum likelihood (REML) method was used for fitting all the models. In order to check the model assumptions, link tests and partial regression plots were used to assess linear model specification; independence, normality, and homoscedasticity of the residuals were also assessed. Standardized effect sizes were calculated for the overall regression models as Cohen’s *f*^2^ (cf. Selya et al., 2012) and for simple effects as Cohen’s *d* from mixed models contrasts and standard errors, and interpreted according to the standard values: *d* = 0.20 and *f*^2^ = 0.02 for small, *d* = 0.50 and *f*^2^ = 0.15 for medium, and *d* = 0.80 and *f*^2^ = 0.35 for large (Cohen, 1992). All analyses were performed using Stata 15.1 (StataCorp, College Station, TX, United States).

## Results

### Sample Description

For reasons not related to the research protocol, three participants missed one administration of the Podium questionnaire assessment (two did not complete the 4th and one did not complete the 5th time measure).

Otherwise, all the data were complete. Sample characteristics presented in Table 1 show that except for their statistically significant differences in height (*p* = 0.001), weight (*p* = 0.003), and BMI (*p* = 0.001), males and females in the sample were otherwise equivalent in athletic performance. Moreover, the two sexes showed almost identical relative marathon performance level around 208% the average of the top 10 marathon runners for each sex. Personal times for both groups were better than typical recreational endurance athletes, since the world’s average finish time of a marathon in 2017 was 4:15:14 for men and 4:47:15 for women (Hanson et al., 2019). No injury occurred during the training or the competition. All runners completed the training according to inclusion criteria and declared to have reached their performance goal in competition. Many of them reached (or were closed to) their personal best time over the distance.

**TABLE 1:** Sample characteristics.

	All	Women	Men	
	Mean (SD)	Mean (SD)	Mean (SD)	*p*
Age (year)	36.9 (4.7)	36.3 (5.6)	37.6 (3.9)	0.576
Weight (kg)	66.8 (14.9)	55.1 (4.5)	78.5 (11.8)	0.001
Height (m)	1.67 (0.1)	1.60 (0.06)	1.73 (0.09)	0.003
BMI (kg/m^2^)	23.8 (3.0)	21.5 (1.8)	26.0 (2.2)	0.001
Endurance training experience (years), median (IQR)	3 (2–4)	4 (2.5–5)	2.5 (2–3.5)	0.257
Personal best marathon time (h, min)	04 h 13 min (38 min)	04 h 17 min (44 min)	04 h 08 min (32 min)	0.668
Relative Marathon Performance level (%)	208.3 (11.8)	208.4 (15.3)	208.1 (21.5)	0.990
HR (bpm) at AeT (Zone 2)	158 (10)	157 (11)	159 (9)	0.716
HR (bpm) at AnT (Zone 4)	176 (9)	177 (9)	175 (9)	0.550
HR_max_ (bpm)	184 (9)	184 (7)	185 (11)	0.810
VO_2__max_ (mL/kg/min)	48.0 (6.4)	45.2 (4.3)	50.8 (7.1)	0.077
MAS (km/h)	14.2 (1.3)	13.6 (1.0)	14.7 (1.4)	0.107
RE testing velocity (% mean marathon speed)	104 (7)	105 (9)	104 (6)	0.618

*BMI = body mass index; Relative Marathon Performance level = runner’s personal best compared to the top 10 best marathon runners in the world; HR = heart rate; bpm: beats per minute; AeT = Aerobic Threshold, determined by Ventilatory Threshold 1; AnT = Anaerobic Threshold, determined by Ventilatory Threshold 2; MAS = Maximal Aerobic Speed, determined by Velocity at VO_2__max_; RE = Running Economy. *p*-values were calculated using two-tailed Student’s *t*-test for independent samples; years of experience were compared using Wilcoxon rank-sum test.*

### Psychological Variables

**FIGURE 1:**
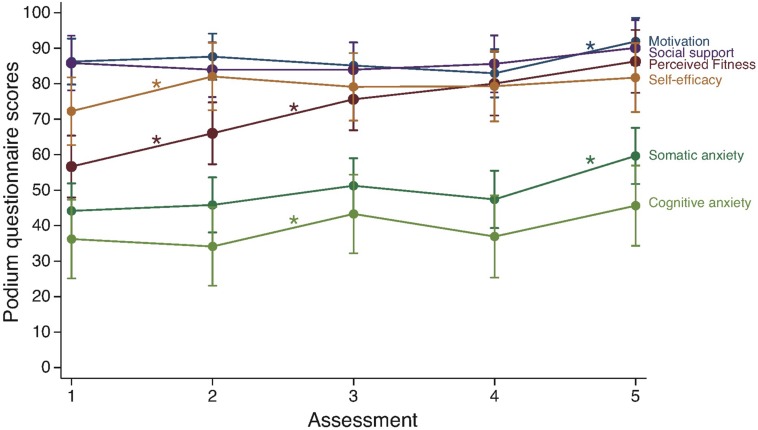
Psychological variables assessed five times over 16 weeks. Assessments were performed at 116, 88, 60, 32, and 11 days prior to competing in the marathon. Data represent mean and 95% confidence intervals adjusted for participant’s random effects. Asterisks represent statistically significant changes (*p* < 0.05).

The adjusted means and 95% CI for each Podium scale over time are shown in Figure 1. Exploratory data analysis showed that the trends seen in the majority of the variables agreed with the reference values proposed by the questionnaire authors (Larumbe et al., 2015). In general, the lowest averages were found at the beginning of the study, and the peak average values were found at the end, except for cognitive anxiety (lowest value at time point 2) and motivation (lowest value at time point 4). Study means over time and reference IQR values for the questionnaire ranged: Perceived Physical Fitness, 56.7–86.3 (reference IQR: 57–80); Self-efficacy: 72.3–81.8 (reference IQR: 63–83); Somatic Anxiety, 44.2–59.7 (reference IQR: 30–57); Cognitive Anxiety, 34.2–45.6 (reference IQR: 27, 50); Motivation, 82.9–91.9 (reference IQR: 70–86); Social support, 83.9–90 (reference IQR: 70–86). Spaghetti plots presenting individual data are included as Supplementary Figure 1.

#### Change Over Time

Only perceived fitness (*p* < 0.001, *f*^2^ = 1.19) and somatic anxiety (*p* = 0.001, *f*^2^ = 0.32) showed statistically significant increases over time associated to large effect sizes. The cumulated effect of time had an initial moderate effect size on perceived fitness (baseline to 2nd measure: Cohen’s *d* = 0.59), and statistically significant large or very large effects until the end of the macrocycle (baseline to 3rd, 4th, and 5th measures: *d* = 1.19, 1.51, and 1.89, respectively, *p* < 0.001 for all). A large effect size compared to baseline was also observed at the end of the study in somatic anxiety (baseline to 5th measure: *d* = 0.99, *p* < 0.001), but only a moderate effect was found at the 3rd measure (*d* = 0.45). Cognitive anxiety only showed a low-moderate cumulated effect at the end of the study (baseline to 5th measure: *d* = 0.41). Self-efficacy showed a modest initial increase that was sustained until the end (baseline to 2nd, 3rd, 4th, and 5th measures: *d* = 0.52, 0.36, 0.38, and 0.51, respectively). Otherwise, the observed effects were small in all variables including motivation and social support.

#### Percent Change

The analysis of percent change between consecutive time points revealed that perceived fitness increased significantly between baseline and time point 2 (34.3%, 95% CI = 16.3–52.4, *p* < 0.001), and between 2 and 3 (19.3%, 95% CI = 1.3–37.4, *p* = 0.035), see Table 2. A similarly high increment was also found in self-efficacy between baseline and time point 2 (39.03%, 95% CI = 11.75–66.3, *p* = 0.005). However, motivation did not show any significant increase between consecutive time points until the last assessment (4.65%, 95% CI = 4.65–26.9, *p* = 0.005). Social support did not show any significant increase between consecutive time points. Both anxiety measures showed and increase at the end of the microcycle, although the change was statistically significant only for somatic anxiety (31.1%, 95% CI = 2.8–59.4, *p* = 0.031). Cognitive anxiety showed a significant increase between time points 2 and 3 (64.5%, 95% CI = 7.8–121.2, *p* = 0.026).

**TABLE 2:** Percent change in study variables between time points (*n* = 16).

	−116 to −88			−88 to −60			−60 to −32			−32 to −11		
	*M*_%dif_	95% CI	*p*	*M*_%dif_	95% CI	*p*	*M*_%dif_	95% CI	*p*	*M*_%dif_	95% CI	*p*
**Podium questionnaire**												
Perceived fitness	34.33	16.31 to 52.36	<0.001	19.35	1.33 to 37.38	0.035	7.45	−11.81 to 26.72	0.448	9.24	−10.75 to 29.23	0.365
Self-efficacy	39.03	11.75 to 66.31	0.005	–2.29	−29.57 to 24.99	0.869	–0.5	−29.67 to 28.66	0.973	10.31	−19.96 to 40.57	0.504
Motivation	3.26	−6.76 to 13.28	0.523	–2.64	−12.66 to 7.38	0.605	0.53	−10.18 to 11.24	0.922	15.77	4.65 to 26.88	0.005
Social support	–2.29	−14.48 to 9.9	0.713	5.59	−6.6 to 17.77	0.369	3.63	−9.4 to 16.66	0.585	8.55	−4.97 to 22.07	0.215
Somatic anxiety	24.62	−0.88 to 50.12	0.058	15.44	−10.06 to 40.94	0.235	–6.98	−34.25 to 20.28	0.616	31.14	2.84 to 59.43	0.031
Cognitive anxiety	28.06	−28.66 to 84.78	0.332	64.51	7.79 to 121.23	0.026	13.12	−45.59 to 71.83	0.661	39.84	−21.09 to 100.76	0.2
**Physiological variables**												
AeTSpeed	3.17	1.57 to 4.77	<0.001	1.32	−0.28 to 2.93	0.105	2.18	0.47 to 3.89	0.013	1.02	−0.75 to 2.8	0.259
AnTSpeed	3.25	1.38 to 5.13	0.001	1.49	−0.38 to 3.37	0.119	0.05	−1.96 to 2.05	0.962	0.39	−1.7 to 2.47	0.717
REmL	–2.72	−5.96 to 0.52	0.100	1.59	−1.65 to 4.83	0.336	–2.55	−6.01 to 0.92	0.149	–2.94	−6.54 to 0.65	0.108
REkcal	–2.44	−5.43 to 0.54	0.108	1.36	−1.62 to 4.34	0.371	–0.82	−4.01 to 2.37	0.614	–2.63	−5.94 to 0.67	0.119

*Time points are presented as days prior to competing in the marathon. Values represent percent change between time points: Mean, 95% confidence interval (CI), and statistical significance of the estimate. AeTSpeed = aerobic threshold speed (km/h); AnTSpeed = anaerobic threshold speed (km/h); REmL = running economy (mL/kg/km); REkcal = running economy (kcal/kg/km).*

### Physiological Performance Parameters

#### Change Over Time

**FIGURE 2:**
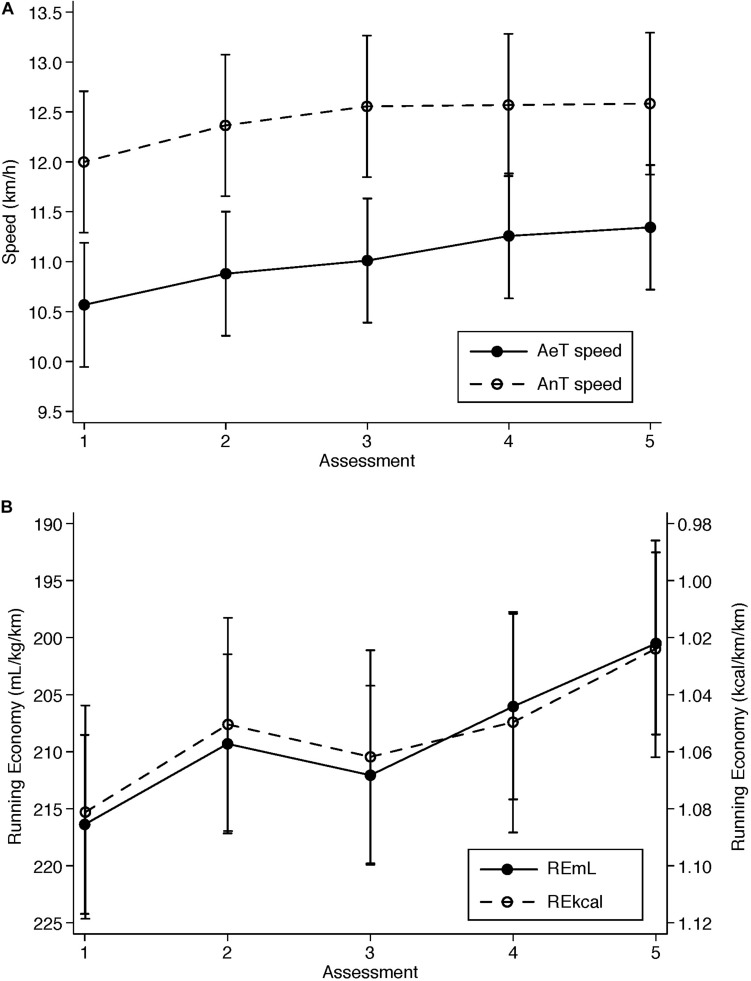
Ventilatory thresholds speeds **(A)** and running economy **(B)** measured five times over 16 weeks. AeT speed: Aerobic threshold speed; AnT speed: Anaerobic threshold speed; REmL: running economy measured as oxygen cost, mL/kg/km; REmL: running economy measured as energy cost, kcal/kg/km. Assessments were performed at 116, 88, 60, 32, and 11 days prior to competing in the marathon. Data represent mean and 95% confidence intervals adjusted for participant’s random effects.

All four physiological variables (Figure 2) presented statistically significant changes over time (AeT speed: *p* < 0.001, *f*^2^ = 1.04; AnT speed: *p* < 0.001, *f*^2^ = 0.498; RE mL: *p* < 0.001, *f*^2^ = 0.349; RE kcal: *p* = 0.024, *f*^2^ = 0.197). Cumulated effects were initially moderate-large in AeT speed (baseline vs. time point 2: + 0.31 km/h, *d* = 0.70, *p* = 0.021) and were progressively larger until reaching a peak at the end (4 vs. 5: + 0.77 km/h, *d* = 1.76, *p* < 0.001). Very similar effects were found in AnT speed both at the beginning (baseline vs. time point 2: + 0.37 km/h, *d* = 0.70, *p* = 0.020) and toward the end (4 vs. 5: + 0.58 km/h, *d* = 1.13, *p* < 0.001). RE mL measures showed moderate or low effects, with a relative fall at the 3^rd^ assessment, from baseline to time points 2 (*d* = 0.47, *p* = 0.216), 3 (*d* = 0.29, *p* = 0.682) and 4 (*d* = 0.47, *p* = 0.032), and reached the effect peak at the end (*d* = 1.07, *p* < 0.001). A similar trend was found for RE kcal from baseline to time points 2 (*d* = 0.45, *p* = 0.265), 3 (*d* = 0.28, *p* = 0.700), 4 (*d* = 0.47, *p* = 0.280), and 5 (*d* = 0.84, *p* = 0.004). Spaghetti plots for physiological variables are included as Supplementary Figure 2.

#### Percent Change

The analysis of percent change revealed that statistically significant increases (see Table 2) between consecutive time points were only found in AeT speed, from baseline to time point 2 (3.17%, 95% CI = 1.57–4.77, *p* < 0.001) and from time point 3 to 4 (2.18%, 95% CI = 0.47–3.89, *p* = 0.013), and in AnT speed, from baseline to time point 2 (3.25%, 95% CI = 1.38–5.13, *p* = 0.001).

### Percent Change Prediction Between Consecutive Time Points

#### Perceived Physical Condition

The longitudinal regression coefficients of the raw data adjusted for time and participants showed that only AeT (6.17, 1.99–10.35, *p* = 0.004, *R*^2^ = 0.14) and AnT (6.42, 2.8–10.04, *p* = 0.001, *R*^2^ = 0.14) speeds were associated with perceived fitness over time, while RE measurement did not show any predictive value.

**FIGURE 3:**
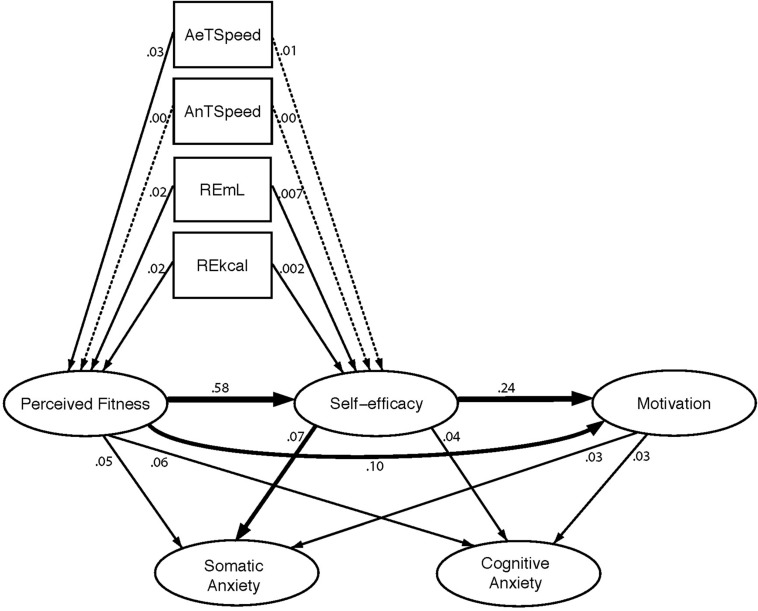
Summary of longitudinal *R*^2^ values found in the study. Numbers represent the effect (*R*^2^) of the predictor adjusted for participant and multiple measurement effects using separate linear mixed effects models for each association tested. Arrows represent the direction of the prediction. Thinness of the arrows represents the magnitude of the effect size (*f*^2^).

However, adjusted for participant and multiple assessment effects, none of the assessed relative changes in physiological variables showed statistically significant predictive value on changes in perceived physical condition. From each independent model, we only found not statistically significant and small effects for AeT speed (*f*^2^ = 0.02), REmL (*f*^2^ = 0.03), and REkcal AeT (*f*^2^ = 0.04); the effect for AnT speed was trivial (*f*^2^ = 0.006). Figure 3 depicts the strength of longitudinal associations examined in the study.

#### Self-Efficacy

Similar to the longitudinal prediction of fitness data, the analysis of self-efficacy raw data coefficients adjusted for time and participants showed that only AeT (7.23, 2.7–11.76, *p* = 0.002, *R*^2^ = 0.04) and AnT (6.56, 2.4–10.72, *p* = 0.002, *R*^2^ = 0.09) speeds were significant predictors, while no predictive value was found in either RE measurement.

Changes in self-efficacy were only predicted by changes in perceived fitness (1.14, 95% CI = 0.88–1.41, *p* < 0.001, *f*^2^ = 1.33) with a statistically significant and very large effect. The prediction of change in self-efficacy from change in physiological performance parameters did not show any statistically significant effect. AeT speed and AnT speed showed a trivial effect size (*f*^2^ = 0.004 and *f*^2^ = 0.001, respectively), and changes in running economy measures showed a small effect size (REmL: *f*^2^ = 0.028; REkcal: *f*^2^ = 0.24).

#### Motivation

Both change in self-efficacy and change in perceived physical condition were significant predictors of change in motivation. However, change in self-efficacy showed a positive large effect (0.19, 95% CI = 0.11–0.27, *p* < 0.001, *f*^2^ = 0.36), while change in perceived fitness showed a positive moderate association (0.21, 95% CI = 0.07–0.35, *p* = 0.003, *f*^2^ = 0.17).

#### Anxiety

Relative increases in self-efficacy were significantly associated with moderate relative decreases in somatic anxiety (−0.32, 95% CI = −0.55 to −0.08, *p* = 0.008, *f*^2^ = 0.13) although the small association with cognitive anxiety (*f*^2^ = 0.04) was not found statistically significant. The association between increases in perceived fitness and decreases in anxiety was found small but statistically significant both for somatic (−0.4, 95% CI = −0.76 to −0.04, *p* = 0.03, *f*^2^ = 0.09) and cognitive (−0.81, 95% CI = −1.6 to −0.02, *p* = 0.044, *f*^2^ = 0.08) anxiety variables. Similarly, small associations were found between increases in motivation and decreases in both somatic (−0.66, 95% CI = −1.32 to −0.01, *p* = 0.048, *f*^2^ = 0.07) and cognitive (*f*^2^ = 0.05) anxiety, although the latter effect was not found statistically significant.

## Discussion

The major contribution of the present study is to depict the evolution of different psychological and physiological variables together in recreational marathon runners over the last 4 months before a marathon race. Since all runners successfully achieved their performance goals at the finish line, the results of this study would characterize the normal evolution of these variables in recreational marathon runners following a systematic training program without major incidents.

Perceived physical fitness increased constantly from an average score of 56.7 at the beginning of the study to 86.3 right before the race. It is difficult to determine a cause-effect relationship between physiological improvements and psychological changes, since multiple covariates might have a role in self-perceiving physical fitness (e.g., experience as a runner, number of marathons run, communication with the coach, comparison to other runners, performance expectations, etc.). Isolating the effect of physiological variables on perception from the effect of other cofounders would be impossible within the present applied research design. Despite this limitation, our findings showed evidence that some systematic improvements in AeT and AnT speeds, but not in RE, were associated to better physical fitness perception. However, when we tried to parallel the changes in both sets of variables we only found not statistically significant small effect sizes.

Despite our results may not be generalized to other runners and training programs, as a practical example, we have found that improving any threshold speed by one km/h was roughly associated to 6% better physical fitness perception. The *R*^2^ value of 0.14 reflected a moderate link that just corroborates previous results (e.g., Delignières et al., 1994; Jensen et al., 2018) (*r* = 0.38) from meta-analytic efforts using other populations (Germain and Hausenblas, 2006). However, percent change analysis between time points did not confirm these results (Figure 3). Therefore, the influence of the physiological performance parameters on perceived fitness should be taken as a long-term relationship rather than an immediate effect.

Average self-efficacy scores were high, ranging from 72 to 82 out of 100. High perception of self-efficacy would reflect the runner’s belief that he or she is ready to perform at the best possible level, while excessive or poor self-efficacy states would lead the runners to be unsuccessful. Therefore, the observed high, but not excessive, self-efficacy scores reflect an optimal mental disposition that should be the norm for runners during the last microcycle and especially right before competing.

Our data partially failed to confirm the hypothesis that improving physical condition would improve self-efficacy. Although the long-term trend showed that improving any threshold speed by one km/h was roughly associated to 6 or 7% better perceived self-efficacy, the analysis of shorter-term relative changes did not confirm these results. However, we did find a strong relationship between perceived fitness and self-efficacy. Approximately, an increase of 10 percent in perceived fitness from any time point to the next was associated with a 11.4% percent increase in self-efficacy during the same period.

Our results showed that motivation remained high during the course of the study, as the average values ranged from 82.9 to 91.9 out of 100. We also found that the sample of runners exhibited also high levels of perceived social support (average range 84–90 during the study), although this variable is not linked to our hypotheses and may not be reproducible in other groups. A deeper analysis of motivation and social support was out of the scope of this study. However, according to the definition of the scales by the questionnaire authors (Larumbe et al., 2015), the motivation score would reflect the internal feeling of commitment and willingness to run, whereas the perceived social support score would reflect either external support or pressure from the interpersonal environment. Both variables showed high values in our sample, reflecting high internal and external sources of motivation for running. Interestingly, the large association between relative changes in motivation and relative changes in self-efficacy scores confirms that self-beliefs of efficacy play a key role in the self-regulation of motivation (Bandura, 1994). In practice, based on our results, a relative increase around 50 percent in either self-efficacy or perceived physical condition would be necessary to produce a 10 percent increase in motivation.

Both anxiety measures showed relative low levels at the beginning of the study: cognitive anxiety averaged 34.2 and somatic anxiety averaged 44.2 out of 100. Levels close to the race day were 45.6 and 59.7, respectively. Overall, in agreement with the existing literature (e.g., Bandura, 1994), medium-term improvements in perceived fitness, self-efficacy and motivation were associated with improvements (decreases) in anxiety, although the effects were predominantly small. However, we found a shift in somatic anxiety from day-32 to day-11 that has been already linked to training load-derived stress (Rehm et al., 2013), and it is also associated with alterations of the immune system, as well as commonly referred gastrointestinal symptoms (Pugh et al., 2018). Interestingly, the increase in somatic anxiety during the last assessment happened in absence of a significant increase in cognitive anxiety (worries). The results achieved in our sample would reflect that the preparation program developed according to the runners’ expectations.

In summary, the general trend observed in the data, where all variables peaked at the end, would mostly reflect that the last training macrocycle before competing is usually planned and executed to provide the athlete with the most adequate preparation, both physically and mentally. These increments in motivation, social support, perceived fitness, and self-efficacy are usually perceived as positive. However, our data showed that some moderate levels of both somatic and cognitive anxiety should also be expected to be normal as a consequence of an optimal preparation.

Physiological performance parameters, especially RE, failed to predict medium-term changes in either perceived fitness or perceived self-efficacy. There are several arguments to explain these results: (a) the residual (or cumulated) effect of fatigue from the training regimen could have impacted negatively the perception of achieving a better physical condition regardless of the physiological changes that objectively happened on the body; (b) as mentioned above, it is impossible in practice to expose regularly the subjects to the real tasks to be performed (i.e., finishing the marathon) in order to produce the perception of physical improvement over time, and therefore marathon runners’ perceived fitness will always hold a large uncertainty component; (c) looking at RE values in Figure 2B, improvements do not necessarily follow a linear continuous upward trend, but an undulating pattern with ups and downs that make hard to self-assess physical condition; (d) in addition, the magnitude of the relative changes (Table 2) indicates that relative changes in perception have much larger magnitude than relative changes in physiological parameters, which indicates that self-perceptions are more inconsistent and are exposed to multiple sources of variation compared to the analyzed physiological variables.

Additionally, RE does not have a direct impact on the finish time like the pace at Zone 2 does. On the one hand, it is very difficult for the athletes to perceive whether they are economical during a short effort, which is the format how RE is usually measured for practical reasons. The human being cannot perceive when the “fuel reserve tank” is being used until the person is already in that situation, which usually takes 20–35 km of the marathon (Joyner, 1991). On the other hand, since the athletes also train according to physiological references (e.g., heart rate, pace), the intensity zones are easier to recognize and therefore to associate to perceptions of physical fitness and self-efficacy.

Nevertheless, all main three psychological variables (perceived fitness, self-efficacy, and motivation) were strongly associated, and linked according to the theoretical framework. This results also confirms the models proposed by Larumbe Zabala et al. (2009), where self-efficacy, perceived fitness, motivation, and anxiety were part of either positive or negative psychological disposition for running the marathon.

### Limitations

Since our sample size was intended to detect moderate effect sizes around a half standard deviation, smaller but practically significant effect sizes may have not been properly addressed. For that reason, the extension of this study and the addition of larger number of subjects and more diverse samples would significantly enhance the description of the expected normal psychological state of marathon runners along their preparation cycles.

We did not consider the possibility that the analyzed associations could occur based on the cumulated perception of change (e.g., improvement over the season). Although this approach seems plausible, it would have required a different data analysis plan by comparing the cumulated change over time instead of percent change between time points. However, this approach would require setting an arbitrary baseline (e.g., at the beginning of a season, when all runners decide to enroll a training program, etc.), which in practice would have added more limitations than advantages to the study design.

### Practical Applications and Future Studies

The presented data support the use of the perceived fitness scale of Podium questionnaire as a subjective measure of progress during the preparation for a marathon. In view of our results, we suggest sport psychology consultants and coaches using this scale to monitor regularly the perception of physical fitness and contrasting the information with physiological and performance tests, since our results suggest that these two sources of information may disagree. In our practical experience, disagreement between athlete’s perception and objective tests would usually be attributed to an improper coach-athlete communication, lack of objective information about performance, excessive time between tests, non-realistic or inadequate goal setting, excessive comparison against other athletes, incidence of injuries and their recovery processes, or alterations in other psychological variables.

For future research regarding the Podium questionnaire variables, we suggest to examine the psychological response of runners to different situations, in order to characterize different profiles and suggest interventions. More specifically, further investigation should examine a cause-effect framework on the dynamics over time of all combinations of excess and/or deficiency in commitment, feeling of preparedness, and anxiety response. It should be expected these states to affect the responses of the subjects at different time point assessments. Consequently, these different response profiles should be associated to training and personal circumstances, in order to attribute the most probable causes for each profile and determine the most appropriate interventions.

Since only non-injured athletes participated in the study, physical performance and its perception were not affected by the occurrence of injuries. Therefore, further studies should explore the effect of injuries on perception of physical fitness, self-efficacy and anxiety levels, using preferably a longitudinal approach.

## Data Availability Statement

The datasets generated for this study are available on request to the corresponding author.

## Ethics Statement

This study was carried out in accordance with the recommendations of the European University of Madrid Ethics Committee with written informed consent from all subjects. All subjects gave written informed consent in accordance with the Declaration of Helsinki. Ethical approval was granted by the European University of Madrid, Spain with number CIPI/035/15.

## Author Contributions

EL-Z and JE-L contributed to the conception and design of the study. JE-L, CC, and AA performed the field assessments. CC organized the database. EL-Z performed the statistical analysis and wrote the first draft of the manuscript. EL-Z, JE-L, and AQ wrote sections of the manuscript. All authors contributed to the manuscript revision, read, and approved the submitted version.

## Conflict of Interest

The authors declare that the research was conducted in the absence of any commercial or financial relationships that could be construed as a potential conflict of interest.

## Supplementary Material

FIGURE S1 Spaghetti plots of Podium questionnaire variables during the course of the study. Each line represents one study participant.

FIGURE S2 Spaghetti plots of physiological performance parameters during the course of the study. Each line represents one study participant.
