# Supplementary figures and images for: Longitudinal Analysis of Marathon Runners’ Psychological State and Its Relationship With Running Speed at Ventilatory Thresholds
Source: Front Psychol. 2020 Mar 27;11:545. doi: 10.3389/fpsyg.2020.00545 (PMC7118226; doi:10.3389/fpsyg.2020.00545)

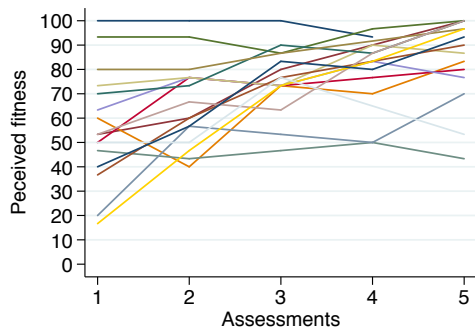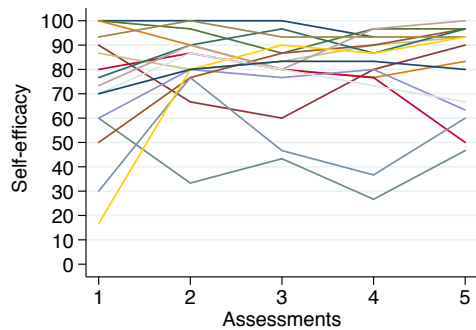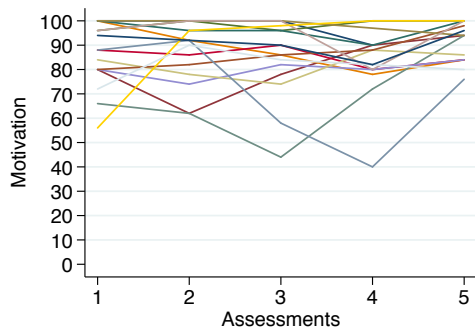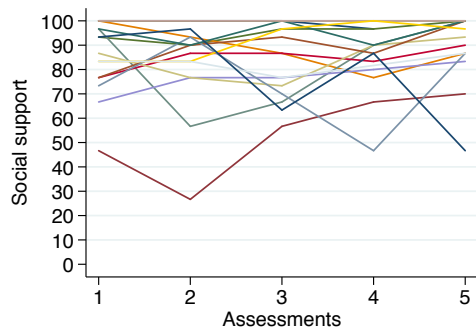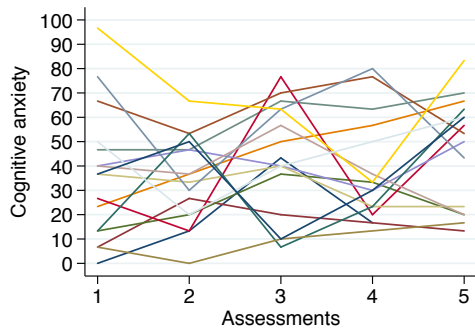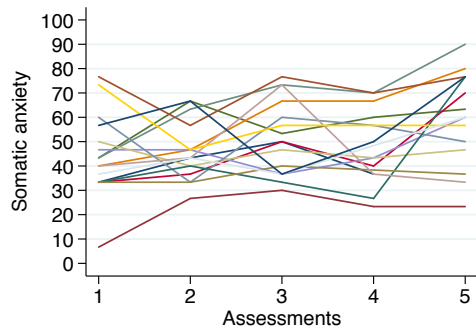

Supplement: FIGURE S1 — Spaghetti plots of Podium questionnaire variables during the course of the study. Each line represents one study participant. [file Data_Sheet_1.PDF]

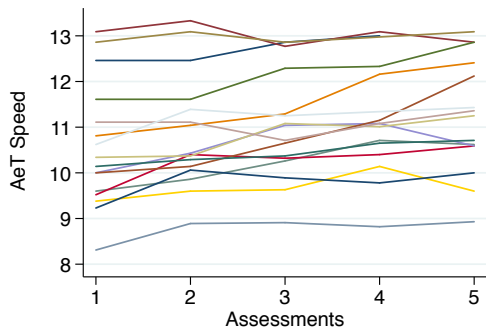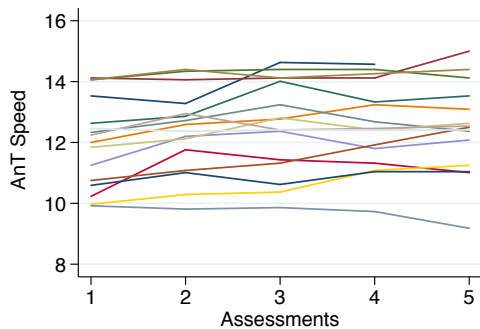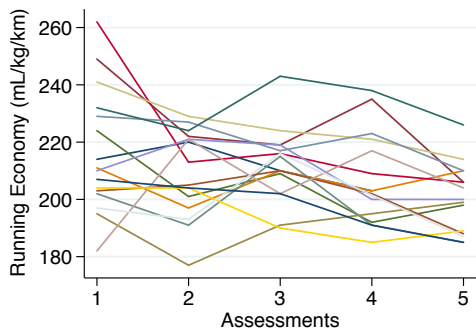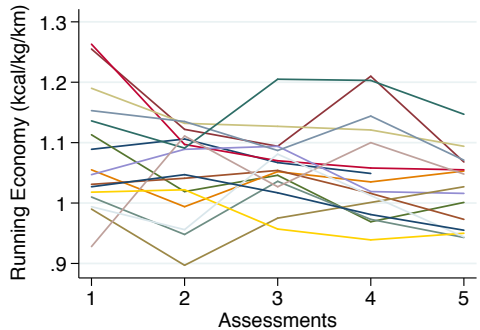

Supplement: FIGURE S2 — Spaghetti plots of physiological performance parameters during the course of the study. Each line represents one study participant. [file Data_Sheet_2.PDF]
